# ‘It is good to have a target in mind’: qualitative views of patients and parents informing a treat to target clinical trial in juvenile-onset systemic lupus erythematosus

**DOI:** 10.1093/rheumatology/keab173

**Published:** 2021-02-25

**Authors:** Eve M D Smith, Sarah L Gorst, Eslam Al-Abadi, Daniel P Hawley, Valentina Leone, Clarissa Pilkington, Athimalaipet V Ramanan, Satyapal Rangaraj, Arani Sridhar, Michael W Beresford, Bridget Young

**Affiliations:** 1 Institute of Life Course and Medical Science, University of Liverpool; 2 Department of Paediatric Rheumatology, Alder Hey Children’s NHS Foundation Trust; 3 Department of Health Data Science, University of Liverpool, Liverpool; 4 Department of Rheumatology, Birmingham Children’s Hospital, Birmingham; 5 Department of Paediatric Rheumatology, Sheffield Children’s NHS Foundation Trust, Sheffield; 6 Department of Paediatric Rheumatology, Leeds General Infirmary, Leeds; 7 Department of Paediatric Rheumatology, Great Ormond Street Hospital, London; 8 Bristol Royal Hospital for Children & Translational Health Sciences, University of Bristol, Bristol; 9 Department of Paediatric Rheumatology, Nottingham University Hospitals, Nottingham; 10 Leicester Children’s Hospital, University Hospitals of Leicester NHS Trust, Leicester; 11 Institute of Population Health, University of Liverpool, Liverpool, UK

**Keywords:** juvenile-onset systemic lupus erythematosus, treat to target, patient perspectives, qualitative

## Abstract

**Objective:**

We sought to explore patient and parental views on treatment targets, outcome measures and study designs being considered for a future JSLE treat-to-target (T2T) study.

**Methods:**

We conducted topic-guided, semistructured interviews with JSLE patients and parents and analysed the audio recorded interviews using thematic approaches.

**Results:**

Patients and parents differed regarding symptoms they felt would be tolerable, representing ‘low disease activity’. Patients often classed symptoms that they had previously experienced, were ‘invisible’ or had minimal disruption on their life as signs of low disease activity. Parents were more accepting of visible signs but were concerned about potential organ involvement and symptom severity. Overall, patients and parents preferred that children were entirely asymptomatic, with no ongoing treatment side effects. They regarded fatigue as particularly challenging, requiring proper monitoring using a fatigue patient-reported outcome measure. Most families felt that reducing corticosteroids would also be a good treatment target. Overall, families liked the concept of T2T, commenting that it could help to improve disease control, help structure treatment and improve communication with clinicians and treatment compliance. They were concerned that T2T might increase the frequency of hospital visits, thus impacting upon schooling, parental employment and finances. Families made suggestions on how to modify the future trial design to mitigate such effects.

**Conclusion:**

This study provides guidance from patients and parents on T2T targets and study designs. Complementary quantitative studies assessing the achievability and impact of different targets (e.g. lupus low disease activity state or remission) are now warranted to inform an international consensus process to develop treatment targets.

**Rheumatology key messages**

- Families felt that the T2T approach may improve disease control, help structure treatment and improve communication and compliance.
- A target stipulating ‘no ongoing JSLE symptoms or treatment side effects’ is preferred by families.
- Patients detest corticosteroids; glucocorticoid toxicity outcome measures are needed to capture reductions in glucocorticoid toxicity.

## Introduction

SLE is the archetypal multisystem autoimmune disorder. Compared with adult-onset SLE, JSLE is more aggressive, with greater disease activity and medication burden, more severe organ manifestations and a higher incidence of renal, cardiovascular and neuropsychiatric involvement [1–4]. Over the past 50 years, 10 year survival has significantly improved from 63 to 91%, although rates have now plateaued [5]. Standardized mortality rates remain higher in lupus than those of the general population, being 18.3 in JSLE and 3.1 in adult-onset SLE [6]. JSLE patients are at high risk of developing permanent organ damage [7] and have significantly lower health-related quality of life (HRQOL) than healthy children/young people, with higher disease activity and organ damage being associated with lower HRQOL [8]. Treatment goals must therefore be ensuring survival, preventing organ damage and optimizing HRQOL by controlling disease activity and minimizing comorbidities and drug toxicity.

Treat to target (T2T), in which treatment is adjusted or escalated until a specific target is achieved, is now part of routine clinical care in many areas of medicine (e.g. RA, hypertension, diabetes) [9]. Development of a JSLE T2T study is one of the key UK research priorities identified within the National Institute for Health Research Clinical Research Network: Children/Versus Arthritis UK Paediatric Rheumatology Clinical Studies Group clinical research strategy [10]. International efforts to develop a JIA T2T study are already under way [11]. A future T2T clinical trial offers a genuine opportunity to substantially reform the clinical management of JSLE patients, using existing treatments in a targeted way with the aim of aggressively controlling disease activity at an early stage, preventing organ damage and helping improve HRQOL.

Development of an adult SLE T2T study has progressed significantly, with a multidisciplinary international task force publishing T2T recommendations in 2014 [12]. Consensus treatment target definitions have been developed and investigated in several SLE cohorts [13–20]. The lupus low disease activity state (LLDAS) is one of the most promising targets, based on the principle of ‘tolerated’ levels of disease activity in a patient on stable treatment and a low dose of corticosteroids [21]. The definition of remission has been extensively debated, leading the Definition of Remission in SLE (DORIS) international consensus group to develop basic principles to which a definition of remission should adhere [22]. Reaching a state of LLDAS or remission has been shown to be associated with less damage accrual [14–18, 23, 24], reduced flare frequency [25], glucocorticoid sparing [20, 25] and improved HRQOL [13]. In adult SLE, it has been proposed that LLDAS and remission could both be used as targets in a T2T study, representing short- and longer-term targets [26].

The Targeting disease, Agreeing Recommendations and reducing Glucocorticoids through Effective Treatment in LUPUS (TARGET LUPUS) research programme has been established in order to develop a future JSLE T2T clinical trial. To this end, it is important to define appropriate treatment targets, treatment strategies to achieve these targets and the most appropriate design for the T2T study. There is currently no guidance on JSLE patient and parental views on the concept of T2T. Therefore the current study aims to explore in depth the views of JSLE patients and parents on potential treatment targets (e.g. LLDAS), outcome measures (e.g. HRQOL and fatigue measures) and study designs being considered by TARGET LUPUS in light of their previous treatment and care.

## Methods

We adopted a qualitative approach involving semi-structured interviews to help inform the development and design of the future proposed JSLE T2T clinical trial. A National Health Service (NHS) Research Ethics Committee approved this study (18/LO/2103), which was conducted and reported in accordance with the Standards for Reporting Qualitative Research [27]. Data from this study are available upon request.

### Participant recruitment

Eight UK paediatric rheumatology centres participating in the UK JSLE Cohort Study [7] served as participant identification sites, facilitating referral of patients who achieved the following eligibility criteria: fulfilling at least four ACR classification criteria for SLE, age ≤18 years, ability to assent or consent and understand spoken and written English. Parents/guardians also had to fulfil the following criteria: capacity to consent for themselves (and/or their child) and understand spoken and written English. Doctors or research nurses invited eligible patients who had previously agreed to be contacted about future studies to participate. Families were approached during routine hospital clinic appointments or over the telephone. The study was briefly described and an age-appropriate participant information sheet, consent form and expression of interest (EOI) form were provided. Families returned their EOI form to the TARGET LUPUS research team and were subsequently contacted by the qualitative researcher (S.G.). Interviews took place between March 2019 and January 2020, mainly in the participants’ homes, and were audio recorded and transcribed. The study team monitored sampling characteristics (gender, age, ethnicity, treatment centre) to ensure that a range of patients was included. Sampling for interviews ceased when data saturation was reached and further interviews were no longer contributing new information [28].

### Interviews

After seeking consent, an experienced qualitative researcher (S.G.) conducted semi-structured interviews using separate topic guides for children, young people and parents (see Supplementary Data S1–S3, available at *Rheumatology* online). Topic guides included five subsections exploring the initial diagnosis of lupus, how patients feel when they are ‘well’ and ‘unwell’, their views on existing definitions of LLDAS, preferences on measurement instruments that aim to quantify their experience of HRQOL and fatigue, experiences of medical treatment and their views on the concept of T2T. A standard description of the concept of T2T (communicated in a conversational style rather than read word for word) was used to help families understand the concept of T2T (see Supplementary Data S4, available at *Rheumatology* online). A diagram illustrating what a hypothetical T2T study might look like (Supplementary Fig. S5, available at *Rheumatology* online) was also used when seeking their views on a T2T trial. Topic guides were adapted throughout the study, guided by the ongoing analysis.

### Analyses

The analysis of interview transcripts drew on a mixture of inductive and deductive thematic approaches, using NVivo 12 (QSR International, Chadstone, VIC, Australia) to assist with data indexing and coding. S.G. coded all interview transcripts, with E.S. and B.Y. reading all transcripts and meeting with S.G. regularly to develop and refine the analysis. M.W.B. reviewed the analyses at a more advanced stage. The analyses followed an iterative process to identify patterns within the data. This involved gaining familiarity with the data by reading and rereading transcripts and creating summaries of each interview; generating initial codes; systematically organizing the data within these codes and comparing across interviews to identify preliminary themes; reviewing, modifying and developing these preliminary themes; and refining the themes [29]. Tables 2–5 provide illustrative quotes with associated quote identifiers (e.g. Q1). Participant quotes are indicated (C, child; P, parent) with the patient number (see Table 1). Patients’ ages are provided (e.g. 16 y). Ellipsis (…) indicate omitted text and square brackets indicate explanatory text. Throughout the results we indicate where the views of patients and parents differed, while generally referring to ‘families’ when both patients and parents were in agreement.

**Table 1:** Participant demographics

Patient number	Gender	Age at diagnosis (years)	Age at interview (years)	Hospital	Parent	Ethnicity	Current treatment	IMD decile^*^
1	Male	11	16	A	Father	Asian	MMF, HCQ	3
2	Female	11	17	A	Mother	White	MMF, HCQ	7
3	Female	7	11	B	Mother	White	MMF, HCQ	7
4	Female	6	11	B	Father	Asian	MMF, HCQ	1
5	Female	13	18	A	Father	White	MMF, HCQ	9
6	Female	16	18	A	Mother	White	MMF, HCQ, Pred	1
7	Female	8	9	C	Mother	White	MMF, RTX	6
8	Female	13	14	D	Mother	Asian	HCQ, MTX	8
9	Female	7	12	E	Mother and father	Black African/ Caribbean	MMF, HCQ	4
10	Female	6	14	E	Mother	Black African/ Caribbean	MMF, HCQ	4
11	Male	11	15	D	Mother	Caucasian	MMF, HCQ	10
12	Female	7	12	F	Father	Asian	MMF, HCQ	10

Two hospitals included in the study did not manage to recruit patients before the recruitment deadline.

Pred: prednisolone; RTX: rituximab.

* Index of Multiple Deprivation (IMD) deciles: 1–3 (high deprivation), 4–6 (moderate deprivation), 7–10 (low deprivation). The IMD score is a widely used indicator of socio-economic background in England [30].

**Table 2:** Quotes to illustrate ‘Finding the right treatment to get lupus under control and feel well’ and ‘Considering “low disease activity state” as a treatment target’

Quote no.	Quote
Finding the right treatment to get lupus under control and feel well	
Q1	‘The first night, I could cross my legs again and everything, then it only took a matter of months to get to 100% almost’. (C2_17y)
Q2	‘One year since diagnosis I was having all this hair loss and rashes. It then disappeared, just went and never came back’. (C1_16y)
Q3	‘Obviously, because you have the side effects and things like that, you do tend to feel worse before you feel better’. (C5_18y)
Q4	‘When she started the treatment, all her complexion changed, weight, everything…She was quite okay going to school normal and everything. But then after a while, it started flaring up again’. (P10_14y)
Q5	‘I’d say quite quickly [felt an improvement] because she was on very high amounts of steroids in the beginning. That took a lot of the symptoms away which then made her able to walk and to function’. (P7_9y)
Q6	‘It’s only probably the last year [3 years post-diagnosis] we seem to have got the medication just right for everything’. (P3_11y)
Perceptions of tolerable symptoms representing a low disease activity state	
Q7	‘[joint pain, muscle aches/weakness and rash] I would say that they were low, because even I have them. Everyone with lupus experiences something like that’. (C5_18y)
Q8	‘The joint pains. Those just never go away…Then some days you’re lethargic, but some days you’re alright…sometimes she’ll have the rash even when she’s well…She’ll still feel okay. That doesn’t stop her from having her normal day’. (P9_12y)
Q9	‘Yes [rash], if it comes with a temperature as well, then we just try to monitor with some paracetamol/ibuprofen. If it still doesn’t go down and we notice her being tired or anything and loss of appetite, then that’s the time we have to take her’. (P12_12y)
Q10	‘The joint problem, well your whole life will change. She was in a wheelchair. If you were still at that point then [6-12 months post-diagnosis] I’d say that you’re still really poorly’. (P7_9y)
Q11	‘It depends on how much they’ve got…So if it’s just one or two [ulcers] then it will be low, if it’s their mouth is full or something, then it will be, again, high’. (P8_14y)
One patient’s ‘low activity’ symptom can be another patient’s ‘high activity’ symptom	
Q12	‘I wouldn't say that rashes were particularly normal, because I don’t really get rashes. So people who do have rashes, it’s high intensity, really’. (C5_18y)
Q13	‘I guess it [pain in chest] would be a concern because it might not just be lupus, it might be something else’. (C8_14y)
Q14	‘[chest pain] I’ve had it before but it’s not really bad. It’s not a really big issue, I don’t think’. (C4_11y)
Q15	‘Yes, mild. Kids get high temperatures’. (P5_18y)
Visibility can make symptoms ‘high activity’	
Q16	‘[Hair loss] is an issue, a big, massive one. [Ulcers] is an issue, especially if it’s on the nose because people might be able to see them’. (C6_18y)
Q17	‘Well, again, that’s [hair loss], clinically, probably not severe, but to a young teenage person, it’s severe’. (P5_18y)
Q18	‘The main things that you couldn’t tolerate is more like the liver not being stable and the joints perhaps because the liver thing is really worrying and very scary…Everything else you can live with in a way over six months but ideally you wouldn't want to’. (P7_9y)

**Table 3:** Quotes to illustrate ‘Experiences of corticosteroid treatment’

Quote no.	Quote
Q1	‘The effect was immediate. When I was on a high dose of prednisolone, I took it and it just started going. The rash started going down. The joint pain, that started going away’. (C1_16y)
Q2	‘I just wanted to come off them. Even when I was only on half a tablet, I didn’t feel happy with being on them’. (C2_17y)
Q3	‘She hated it. Because it was the side effects. Weight gain. What was your face, bloated face. Increased appetite, wasn’t it?’ (P6_18y)
Q4	‘I wish that I hadn’t really done the steroids at all, because I don’t think I really needed them…It didn’t really do anything for me…I probably didn’t really need the steroid injection and the tablets’. (C5_18y)
Q5	‘I just think it was the word ‘steroids’. She just thought, ‘Steroids are going to make me big and fat’…I think it stabilised her for a short time, and gave time for the lupus treatment to start to kick in’. (P5_18y)
Q6	‘My mum used to tell me that obviously you’re not meant to have steroids for a long time because it’s not best for you, so I used to cry and think, ‘Oh no, I’ve been on them too long’ and stuff’. (C8_14y)
Q7	‘Yes. The less time you’re on them, I think the less time your body relies on them as well is better’. (C7_9y)

**Table 4:** Quotes to illustrate ‘Strength and weaknesses of health-related quality of life/fatigue measures**’**

Quote no.	Quote
Q1	‘The questions [on the Rheumatology Module] were definitely relevant’. (C8_14y)
Q2	‘I would really feel comfortable in filling out this one…because if she had these things then going to school, mixing with children, she would never, you know if she’s in pain and all these things’. (P10_14y)
Q3	‘I like that one [Rheumatology Module] the most. That one is a good one. I think that is more about the actual lupus and then I guess that’s [PedsQL Inventory] about your mental health. They are both about different things, so they both go their own ways’. (C8_14y)
Q4	‘It’s the best one because it’s easy answering with the faces…They are easy to understand’. (C4_11y)
Q5	‘That question’s a bit stupid because having lupus doesn’t make me feel happy. But it doesn’t make me feel sad…I wouldn’t say how lonely do you feel because of lupus. Like, I’m not sad, I’m not lonely, but I’m not happy’. (C6_18y)
Q6	‘It is important, because fatigue is a main issue when you’ve got lupus, anyway, isn’t it?’ (P11_15y)
Q7	‘Fatigue in itself when they’re struggling with it is a worrying part of it for a parent. Kids have huge energy levels. When that’s gone and they are suffering with fatigue, it’s really worrying. For doctors to take that aspect seriously is a good thing, from our point of view’. (P7_9y)

**Table 5:** Quotes to illustrate ‘Acceptability of treat to target’

Quote no.	Quote
Q1	‘Yes, so if you’re having these frequent visits in the first year, and then people know what’s going on, everyone knows what you’re working towards…you get to learn the disease better, to understand the treatments’. (P9_12y)
Q2	‘The treat to target one is better because every month they can check on you so there’s no risk. In the three months one, there are three months without checking on you…for a new patient it would be better because they have a better chance of having a good experience in life’. (C4_11y)
Q3	‘If I was newly diagnosed, I wouldn’t be very happy with [routine care], especially with the gaps between the visits. The fact there’s no targets means you won’t have an aim before and after you go to an appointment. Compared with this T2T, this routine care isn’t very good’. (C1_16y)
Q4	‘I just think it would be better to have a target, because it means more communication between you and the clinic, basically’. (C5_18y)
Q5	‘Because it just shows that you’re getting better, so you don’t really need them as much, and you can just cut them down and then eventually stop having them’. (C11_15y)
Q6	‘Okay, I should take this medicine because after a week or so I have to give the blood test, or something’. So I think it will make children punctual, themselves, to get medicine’. (P8_14y)
Q7	‘You know, your time off work, it’s paying for your car parking, all your other kids and stuff. So, that’s always on your mind, as well as them doing all this’. (P2_17y).
Q8	‘It is good to have a target in mind, but if they don’t reach it by a certain time, you shouldn’t push it. It is likely to be reached’. (C2_17y)
Q9	‘Maybe that [treat to target] would be a bit too confusing and stressful for the patient, because if you’re continuously changing the medicines, they might start thinking that something is wrong with them and the medicine is not working on them…so maybe they have to give it more time for them to reach the target’. (C8_14y)
Q10	‘I struggle to see with lupus because there are so many different areas…You could just be treating some symptoms, but not the actual issue’. (P2_11y)
Q11	‘I think, maybe, monthly might be a bit short…but maybe a six-week thing…because it’s just a little bit extra time…I think it’s just important to give the medication time’. (C5_18y)
Q12	‘For three to six months, they’ll go monthly and then I think once they come into a routine medication, they just need to go the standard three months…Once they’re settled, yes’. (C8_14y)
Q13	‘I mean with the T2T, I absolutely agree it’s probably a better way of going about things than just the normal routine care as long as obviously people are aware…, which doctors will be, and parents are made aware that things can change and they change very quickly’. (P7_9y)
Q14	‘They should let everyone have the chance [to be treated to target]’. (C4_11y)

## Results

### Participants

A total of 44 families were approached to participate in the study and 22 EOI forms were received (50% response rate). Of these, three later declined, three could not be contacted and four families returned their EOI form after recruitment had ended. Twelve families were interviewed, comprising 12 patients and 13 parents (Table 1). Patients were between 9 and 18 years of age (median 14 years) at the time of the interview and were treated at six UK hospitals. They had been diagnosed with JSLE at 6–16 years of age (median 10 years). Interviews were held in either the participants’ homes (*n* = 10 families) or in a private room at their hospital (*n* = 2 families). Patients and parents were interviewed individually during half of the interviews. However, parents remained in the same room during six of the interviews. Patient interviews lasted 22–58 min (median 37 min), while parental interviews lasted 23–91 min (median 60 min).

### Qualitative results

#### Finding the right treatment to ‘feel well again’ and treatment side effects

Patients gave varying reports of the pattern of their initial recovery after the JSLE diagnosis and how long it took to feel well. Some noted that initial treatment quickly relieved some of their overt difficulties (e.g. severe aches and pains), although it took longer to feel properly well again (Table 2, Q1). For others, the initial improvement was slow and then they quickly felt well again (Q2). Some patients linked a delay in feeling well to treatment side effects they experienced (Q3). Some parents noted that their child experienced ‘peaks and troughs’ in disease activity (Q4) before things came under control. Several parents attributed initial improvements to high-dose corticosteroids (Q5), a treatment that one parent described as ‘more of a band aid’. Other parents noted trying several treatments before their child’s disease was controlled (Q6). No families reported early discussions with medical staff about working towards a predefined target that took account of disease activity, treatment side effects and the impact of lupus on their life.

#### Considering ‘low disease activity state’ as a treatment target

##### Perceptions of tolerable symptoms representing a low disease activity state

Patients and parents both tended to mention joint pain, rash, muscle aches and/or weakness when asked what symptoms they would class as low disease activity (Table 2, Q7). Families tolerated symptoms that did not disrupt a child’s life and could be controlled most of the time (Q8). However, several minor symptoms appearing simultaneously were more concerning (Q9). Parents of children who had experienced disabling arthritis regarded joint pain as indicating high disease activity (Q10). Many families commented that it was not the presence of a symptom, but its severity, that influenced whether they would class it as low or high disease activity (Q11).

### One patient’s ‘low activity’ symptom can be another patient’s ‘high activity’ symptom

Patients often classed symptoms that they had not previously experienced as signs of high disease activity (Q12), particularly if the symptom was associated with a potentially serious illness. For example, chest pain was infrequently experienced but was regarded as a sign of high disease activity (Q13), probably because chest pain potentially signifies a heart attack in adults. However, if a child had previously experienced chest pain, families were less concerned about its occurrence (Q14). Despite only a few patients experiencing fever as part of their lupus, most families were not concerned, as they believed it represented a common, self-limiting illness (Q15).

### Visibility can make symptoms ‘high activity’

Adolescent patients had little tolerance for visible symptoms such as alopecia, rash and mouth/nose ulcers (Q16). In contrast, parents tended to describe visible symptoms as low disease activity, while recognizing their significance for their child (Q17). In the context of having had a child with severe internal organ involvement, parents often accepted visible symptoms that they felt would not lead to organ damage (Q18).

#### Experiences of corticosteroid treatment

All patients had received systemic corticosteroid treatment at some point but were conflicted in their views towards corticosteroids. Many noted their effectiveness in treating lupus symptoms (Table 3, Q1). However, there was strong dislike of corticosteroid side effects (Q2). Patients and parents alike noted the worst side effects to be weight gain, bloated face and increased appetite (Q3). Some patients regretted taking corticosteroids, as they felt their lupus was too mild to warrant such treatment (Q4). In contrast, none of the parents mentioned that the corticosteroids were unnecessary. Rather, parents emphasized how important the corticosteroids had been in helping to reduce the severity of their child’s symptoms soon after diagnosis (Q5). Unsurprisingly, many patients would have liked to reduce corticosteroids quicker than they had and were concerned about taking corticosteroids for an extended period (Q6). Nevertheless, patients and parents agreed that reducing corticosteroids would be a good treatment target (Q7).

#### Strengths and weaknesses of HRQOL/fatigue measures

Participants completed four age-appropriate child and parent proxy-reported HRQOL questionnaires: the PedsQL Rheumatology Module [8, 31], PedsQL Pediatric Quality of Life Inventory [32, 33], Simple Measures of Impact of Lupus Erythematosus in Youngsters (SMILEY) [34, 35] and PedsQL Multidimensional Fatigue Scale [36]. Patients and parents all favoured the PedsQL Rheumatology Module, as they thought it provided the clearest picture of well-being and functioning (Table 4, Q1–2). Some also favoured using it in combination with the generic PedsQL Inventory, commenting that one complemented the other (Q3). Participants were polarized in their views on the SMILEY questionnaire. While popular with younger children who enjoyed circling the SMILEY face response options (Q4), other patients and parents strongly disliked the SMILEY questionnaire, as they found the response options to be confusing (Q5). Almost all patients and parents thought it was important to have a separate questionnaire focussing specifically on fatigue. They regarded fatigue as one of the ‘biggest challenges’ associated with having lupus (Q6) and noted that the questions within the PedsQL Multidimensional Fatigue Scale were of direct relevance to them. Parents additionally emphasized how distressing fatigue can be and the importance of doctors monitoring it properly (Q7).

#### Acceptability of T2T

The concept of T2T can be difficult to understand and thus was explained by showing participants a diagram illustrating what a hypothetical T2T study might look like (Supplementary Fig. S5, available at *Rheumatology* online) and pointed to a potential increase in clinic visit frequency, structured assessments of the target at each visit and increasing the strength or number of treatments at set time points if the target is not achieved.

Views were explored on an exemplar T2T trial design where participants would be randomized to receive T2T or standard care. Almost all families thought T2T would be a preferred treatment approach to standard care. Families thought frequent clinic visits were important for newly diagnosed patients so that they could be monitored more closely (Table 5, Q1–2). They felt that having a target would enable them to have a better understanding of how well treatment was progressing (Q3–4). Families also thought achievement of the target could help when making the decision to reduce or continue a treatment (Q5). Some felt the increased frequency of visits could have a positive effect on treatment compliance (Q6).

Some participants were concerned that more frequent clinic visits in a T2T trial would impact on schooling, parental employment and family finances (Q7). Some families qualified their enthusiasm for T2T with concerns that T2T could lead to unnecessary and rapid increases in treatment (Q8). Similarly, patients were worried about possibly having to change medication frequently if the target is not reached (Q9). Some parents felt that a T2T approach was too simplistic, questioning whether it would be possible to set a treatment target for JSLE patients (Q10).

Families were asked how they would change the proposed study. Some suggested having six weekly clinic visits (Q11), whereas others suggested monthly visits initially, moving to visits every 3 months once medication became stable (Q12). Regarding the treatment target, one parent highlighted the importance of families being made aware of the possibility of rapid treatment changes (Q13). Some families were concerned about the possibility of being randomized and suggested that everyone should have the T2T option available to them (Q14).

**Fig. 1:**
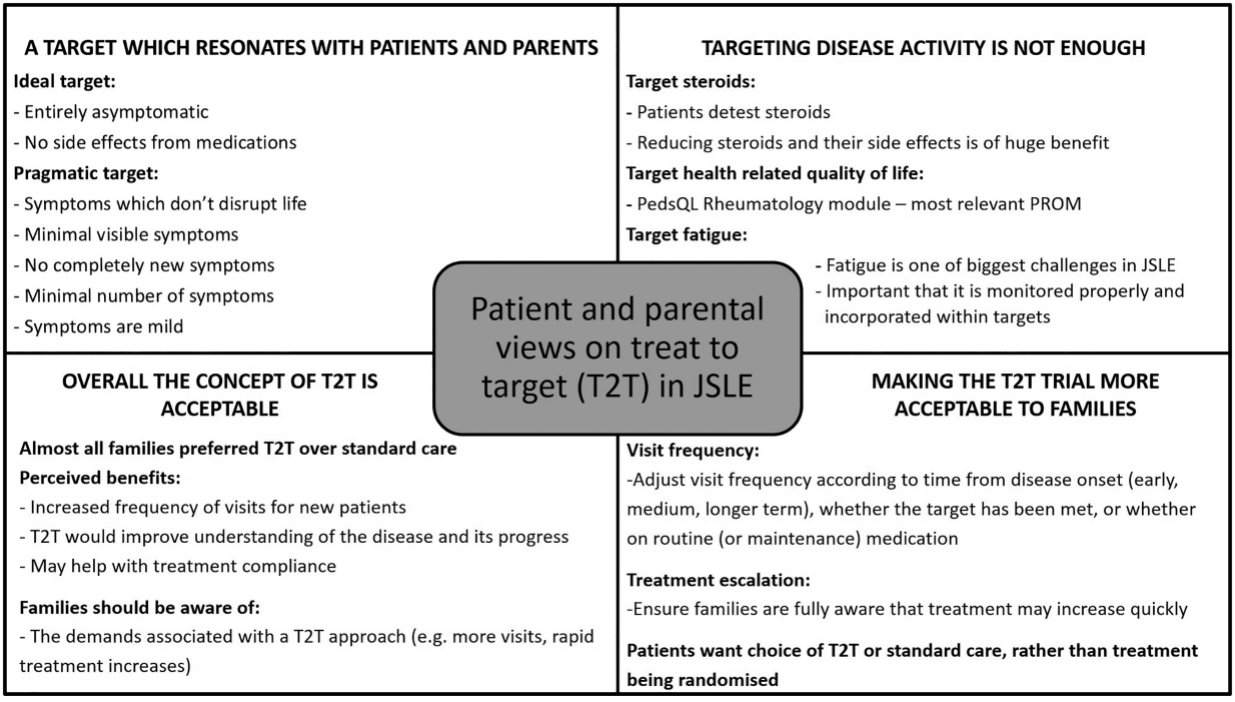
Summary of patient and parental views on T2T in JSLE

A summary of the overall patient and parental views on T2T in JSLE are shown in Fig. 1.

## Discussion

This is the first study specifically exploring the views and perceptions regarding proposed treatment targets, outcome measures and T2T treatment strategies of patients with JSLE and their parents. The study is part of a wider programme that aims to inform the development of a future JSLE T2T clinical trial.

Lupus patients at diagnosis are a heterogeneous group, differing markedly in their presenting symptoms and severity of their disease, and the narratives of the families interviewed reflected this. The interviews revealed noticeable variations in the patterns of recovery and time it took different patients to ‘feel well’, largely due to differences in their response to initial treatment. Some patients responded well to the first treatment and gradually improved until they were feeling back to normal. In comparison, others had more of a rollercoaster experience, which involved trying multiple different treatments until their lupus was under control. This observation emphasizes the importance of timely decision making and, where necessary, treatment escalation to achieve disease control quickly and reduce the impact of lupus on the patient’s life.

A pragmatic approach to a JSLE T2T study could be to aim to reach LLDAS, as remission can be difficult to achieve [37]. Participants were asked which lupus symptoms would they regard as consistent with LLDAS, which we explained as being when a patient’s lupus is not very active but the patient is not completely back to normal. Definitions of LLDAS have been derived in adult SLE [21, 38, 39], but JSLE-specific definitions have not been developed. For adult SLE, patients would be regarded as being in LLDAS if they experience arthritis or myositis or up to a maximum of two of the following: rash, hair loss, mouth ulcers, fevers and chest pain. When families were asked for their views about these symptoms, there were no specific symptoms that they consistently regarded as indicative of low disease activity. Rather, they tended to class symptoms that they had previously experienced as being representative of LLDAS, with the diverse symptomatology of JSLE leading to different combinations of acceptable symptoms in each family. Any LLDAS symptom could be regarded as consistent with LLDAS as long as the symptom was mild and not impacting on the patient’s life. However, adult SLE LLDAS definitions do not specify symptom severity. Adolescents were particularly intolerant of visible signs that made them stand out from their peers. In a previous qualitative study, Tunnicliffe *et al.* [40] described visible signs of SLE as ‘marring the identity’ of young people with SLE, leading them to perceive themselves as ‘sick and incapacitated’ rather than ‘young and healthy’. Such signs led to poor self-image, teasing at school and a sense of isolation, further emphasizing the impact and importance of visible signs to young patients.

Overall, families indicated that their preferred target would be no ongoing JSLE-related symptoms or treatment side effects. In view of this finding, we suggest that a target of remission would be closer to families’ preferences than LLDAS, although further exploratory work is needed to define what remission would entail from the perspective of children and parents. When the SLE T2T task force recently met to reach a consensus definition of remission for adult patients [22] it was noted that ‘several task force members including patient representatives were concerned that the patient’s perspective was not explicitly included in the definition, and emphasized the importance of a definition of remission that “resonates” with the patient’. Our findings from families echo the experience of the SLE T2T task force, whereby the heterogeneity of lupus makes it difficult to develop a definition that suits everyone and further highlights the need for additional research and consensus work dedicated to the topic of remission in its own right. Studies looking at the achievability and impact of achieving existing definitions of target states, such as LLDAS and remission in JSLE, are also warranted to help inform consensus work with both families and physicians.

Important secondary outcome measures in a JSLE T2T trial include patient- and parent-reported outcome measures. When asked to reflect on the suitability of different HRQOL and fatigue-based questionnaires, families identified the PedsQL Rheumatology Module as the most useful for assessing HRQOL, either alone or in conjunction with the PedsQL Inventory. They strongly supported formal assessment of fatigue as part of clinical care and agreed that the PedsQL Multidimensional Fatigue Scale, which has been validated for the assessment of fatigue in paediatric rheumatology populations [36], would be a good secondary outcome measure in a T2T trial.

Despite the SMILEY HRQOL questionnaire being specifically validated in JSLE [34], participants in this study were divided in their opinions on it. The content of the SMILEY was derived from a qualitative research study that involved children and their parents being asked the question: ‘Would you like to say anything else about you/your child having lupus? You may write as little or as much as you want’. Responses were either hand written or spoken and recorded verbatim [41]. The qualitative study by Tunnicliffe *et al.* (mentioned above [40]) involved both semi-structured interviews and focus groups, providing in-depth insights into the experiences of adolescents and young adults diagnosed with JSLE, and demonstrated additional items that are of particular relevance to HRQOL in JSLE and could be considered for inclusion within the SMILEY to improve its relevance to patients, namely ‘knowledge of SLE, confidence in accessing healthcare, and perceived capacity for self-management’.

Glucocorticoid treatment is one of the main risk factors for long-term irreversible damage in JSLE [42]. Glucocorticoid-related organ damage is higher among JSLE than adult SLE patients [43]. Families acknowledged the importance of corticosteroids in quickly treating lupus symptoms but were frequently distressed by the unpleasant side effects, wanting to stop corticosteroid treatment as soon as possible. Most families thought that reducing corticosteroid treatment represented a good T2T target. A glucocorticoid toxicity index (GTI) has been developed to assess the glucocorticoid-related morbidity and glucocorticoid sparing ability of other therapies in adults with SLE [44]. Adult patients’ views on the most important positive and negative effects of glucocorticoids in the treatment of SLE and myositis have also been identified [45]. Aggressive use of corticosteroid-sparing treatment strategies within a T2T clinical trial may be of benefit, but appropriate methods for quantifying glucocorticoid toxicity in children must be developed to capture any associated reduction in toxicity/damage.

Families were accepting of a T2T approach, commenting that it would structure treatment, enable more frequent clinic visits, help to catch complications more quickly and aid compliance with medications. They felt that working towards a common goal (the target) may improve communication between the doctor and families and help to reduce treatment (e.g. corticosteroids) when things are improving and the target is achieved. However, some families were concerned about the impact of increased visits on other aspects of their lives (e.g. schooling, parental employment and finances). Families suggested modifications to the frequency of clinic visits. Some parents questioned whether it would be possible to set a target for JSLE patients due the complexity of the disease and were apprehensive about the potential need for treatment escalation. Tunnicliffe *et al.* [40] described the theme of ‘animosity towards medication use’, with one patient voicing that at times they ‘felt like a guinea pig because of constant switching of medicines’. Taken together with the results of this current study, this highlights the crucial need for shared decision making, careful communication and patient and parental involvement when developing a T2T clinical trial, particularly in relation to the study design, recruitment and study implementation.

A recent commentary discussed the patient perspective on T2T in RA, PsA and JIA [46]. Overall, the authors felt that patients were accepting of a T2T approach and liked the principle of ‘tight disease control’. However, they commented that such an approach would not significantly improve outcomes if there was a single target based solely on disease activity or ‘abrogation of inflammation’. Instead, they proposed that there should be several goals in addition to disease activity, including HRQOL, pain, fatigue and function, and that goals should be individualized and agreed upon with patients through a shared decision-making process. They also discussed that ‘treatment’ should not be limited to medications but should also include interventions that could address the goals described above (e.g. physical therapy, specialized surgery, psychological support). The authors also highlighted divergences between clinicians and patients when using the term remission (as discussed above), with clinicians aiming for ‘biological remission’ and patients preferring a more holistic definition of remission, including inflammation as well as disease impact. These perspectives are largely in keeping with the findings of the current study, whereby families wanted to minimize disruption from JSLE in all aspects of the patient’s life and preferred treatment goals to include corticosteroid dose reduction, HRQOL and fatigue in addition to targeting of disease activity.

While interviewing ethnically and socio-economically diverse families from six UK hospitals, we acknowledge important study limitations. Only 2/12 (17%) patients were male, although this reflects the demographics of JSLE [47, 48]. Five of the 12 patients had early-onset JSLE (<8 years old), with the potential for more severe disease and a stronger genetic contribution to their disease. However, while the sample may overrepresent patients with severe disease, patients with less severe illness were also included and, as such, the study captures the perspectives of patients across the illness severity spectrum. Most patients had been diagnosed with lupus >4 years ago and many reported feeling very well at the time of the interview. This may explain why families were generally reluctant to accept ongoing symptoms. When the qualitative researcher visited patients’ homes, she always asked if she could interview patients separately from their parents. However, some patients preferred their parents to stay with them, and in some houses there was no separate area where parents could sit and wait while their child was being interviewed. The interviews did not involve asking patients about any particularly sensitive issues, so there was no concern about patients not speaking in front of their parents. Finally, we were encouraged that there were numerous instances of patients and parents expressing different views.

The concept of T2T is complicated and hard to explain and some families found the concept difficult to understand. Therefore the concept of T2T evolved iteratively over the course of the study to help patients and parents better understand what the target might look like, how it would be assessed and the process of escalating treatment in a structured way if the target is not met. Most families were able to communicate their opinions on T2T, detailing positive and negative aspects and providing suggestions to improve the study design. Nevertheless, patient engagement work is needed to refine how best to communicate the concept of T2T to patients of different ages and parents. An accessible explanation of the concept will be important during recruitment to a T2T clinical trial and will help the research nurses and clinicians when approaching potential patients and seeking consent.

## Conclusion

This study has provided guidance from JSLE patients and parents to assist with the development of a future T2T clinical trial. Most families were reluctant to tolerate ongoing symptoms and opinions varied widely in the particular symptoms they saw as low level or tolerable. Influences on whether a particular symptom was tolerable included whether it disrupted the patient’s life and both the severity and visibility of the symptom. However, overall, families preferred being entirely asymptomatic, with no ongoing treatment side effects, suggesting that remission is a more attractive treatment target for them than LLDAS. Further work is needed to develop a definition of remission that is acceptable to families and align with medical definitions of remission in JSLE. Studies assessing the achievability and impact of achieving either LLDAS or remission in JSLE are warranted to also help inform deliberations with families about potential treatment targets. Patients and parents agreed that reducing corticosteroids is an important treatment target. They favoured having both HRQOL- and fatigue-specific secondary outcome measures within a T2T trial. The concept of T2T, aiming to improve disease control, structure treatment and improve communication and compliance, is acceptable to families. Families welcomed the increased frequency of hospital visits after the initial diagnosis. They suggested spacing visits out once things are more stable to reduce the impact on schooling, parental employment and family finances. The findings will inform future international consensus meetings focussing on development of the T2T clinical trial to ensure that the perspectives of both patients and families are considered alongside JSLE healthcare experts.

## Supplementary Material

keab173_Supplementary_Data
