# Supplementary material for: ‘It is good to have a target in mind’: qualitative views of patients and parents informing a treat to target clinical trial in juvenile-onset systemic lupus erythematosus
Source: Rheumatology (Oxford). 2021 Feb 25;60(12):5630–41. doi: 10.1093/rheumatology/keab173 (PMC8645274; doi:10.1093/rheumatology/keab173)
Supplement: keab173_Supplementary_Data [file keab173_supplementary_data.zip › keab173-suppl_data/rhe-20-2289-File003.docx]

**Topic guide for children’s qualitative interviews**

(aged <12 years)

**‘Living with Lupus’ - developing treatment targets and outcome measures with children and young people to TARGET LUPUS**

NOTE: Qualitative interviews will be conversational and tailored to the individual participant. So, this is only an outline of the topics and questions that will be covered and it will be updated in the light of the ongoing interviews and analysis.

***First section – background and establishing rapport***

*This first bit of the interview is all about YOU and your initial diagnosis of Lupus.*

- What hospital do you go to?
- When were you diagnosed with Lupus?
- What was it like for you when you first started having problems due to Lupus?
- How are you feeling today?

***Second section – exploring what it means to patients to ‘be well’ and comparing this to medical definitions of being in a ‘low disease activity state’***

*This part of the interview is to explore how YOU feel when you are ‘well’ and ‘un-well’?*

- Since being diagnosed with Lupus, has there been a time when you have felt well?
- What is a good day like for you?
- When you feel ‘well’ (or have a good day) does your Lupus go away? If no, you still have a few symptoms or signs of Lupus - what do they tend to be?
- Are there any signs of Lupus that just wont go away, no matter what you or your doctors try?
- Since being diagnosed with Lupus, have you ever felt completely back to normal, what you were like before you had lupus? (Note - here we are trying to get at whether they feel they have been in complete remission)
- Are there any symptoms of Lupus that you don’t even bother mentioning to the doctor/anyone as you don’t think anything can be done about them?
- What is your lupus like when it has been at its worst? What is a bad day like?
- If a Lupus patient was described as being in a ‘low disease activity state’ where their lupus isn’t very active, but they are not completely back to what they were like before they had Lupus, what symptoms / signs of Lupus would you still expect them to have?
- This question relates to the existing definition of LDAS - would you class yourself as ‘not perfect but fairly well’ if you had any of the following symptoms / features of lupus?
  - Arthritis (PROMPT – 2 or more joints with pain or swelling)
  - Myositis (PROMPT - muscle aching or weakness)
  - A rash
  - Alopecia (PROMPT – hair loss)
  - Ulcers affecting you mouth or nose (PROMPT – sores in your mouth)
  - Pain in your chest
  - A fever (PROMPT – temperature of more than 38^o^C)
- When you are ‘fairly well’, considering all the ways that Lupus affects you, how would you rate how you are doing by placing a single mark on this line. SHOW A PICTURE OF THE 0-10 SCALE BELOW.

Very well 0
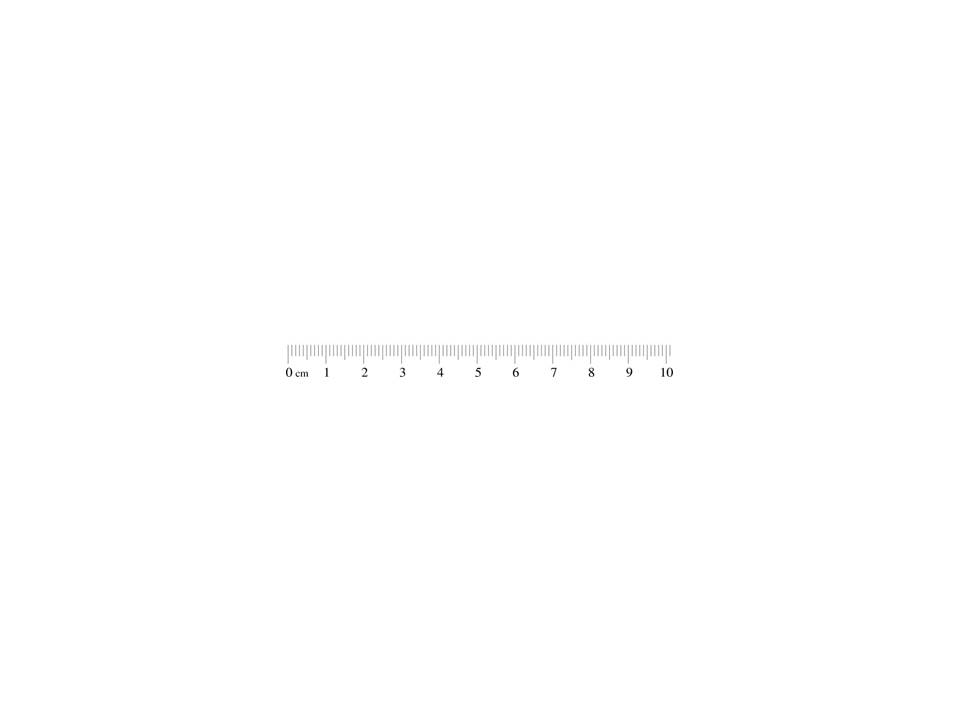
 10 Very poor

***Third section – patient reported outcome measures***

*In this part I would like to ask what you think of different questionnaires and tools that can be used to monitor how you are doing over time. When you are filling in the questionnaire it would be useful if you could say everything that you are thinking out loud, so that Sarah can get an idea of what you think and feel about the different questions. [NOTE – during periods of silence, remind the participant to keep thinking aloud].*

For each tool we are interested in:

**a) respondent burden** (time to complete the questionnaire, the interviewer will record the time it takes to complete the questionnaires without making it too obvious, so as not to put the participant under pressure to complete the questionnaires quickly);

**b) feasibility** (are they able to complete all questions, what proportion of the questions are left unanswered, why are they left unanswered e.g. can’t understand to question, not relevant to them, other);

**c) expected answers when well** (when they are well do they think they would have a perfect score for all items or do they think there are some items where they still wouldn’t score perfectly).

For each questionnaire:

- Can you have a go at filling in this questionnaire.. Try to fill in all of the questionnaire but if there are questions that you don’t understand / can’t fill in, leave them blank and we will discuss them afterwards. Take as long as you like – if you would like me to read the questions out for you then that is not problem. If you want to read them yourself then I will get on with some work in the meanwhile.
- At the end, after completing all questionnaires:
  - Go through any questions left blank or participant found confusing.
  - Thinking back to a time when you have been well [NOTE - refer to the answer at the start if they have said they are feeling well today], how would you have scored all the different bits of the questionnaire. Do you think there are some activities that you would have struggled with, even though you were well?
  - Which HRQOL questionnaire do they prefer, and what makes you prefer that one?
  - Do you think having the fatigue questionnaire is useful?

***Fourth section – experiences of steroid treatment and reflecting on the adult glucocorticoid toxicity index***

*One of the types of medicine that you will have been given is called a steroid (for example prednisolone or the intravenous version which is called methylprednisolone or methyl pred for short). This part of the interview is about those types of treatment.*

- Do you like or dislike steroid treatment? PROMPT – what makes you like or dislike them? Is there anything in particular that you like or dislike?
- What types of steroid have you had in the past? (PROMPTS – oral or into a vein)
- Have you struggled to get the dose of your steroid treatment down?
- Have you had any side effects from steroids?
- What have been the worst side effects you have had from steroids?
- When you have been on a lot of steroid medicine in the past, have you wished that you could come off steroids (or reduce them) quicker than you have been able to?
- Would reducing the steroid dose you are on be a good treatment target?

**Fifth section – exploring patients experience of medical treatment and eliciting some preliminary opinions on a Lupus treat to target study**

In this final section, I would like to find out about the medical treatments you have had, how you have felt about these treatments and the way your care went in the first year after your diagnosis of Lupus.

I would also like to explore what you think about the idea of a medical study that doctors would like to carry out, to find out how children and young people with Lupus should be treated. [Note - Explain/reassure before and afterwards, as necessary, that this is only an imaginary medical study at the moment. We are not actually requesting participation.]

**Exploring the patients experience of medical treatment**

- Your mum/dad tells me you were diagnosed with Lupus (N) years ago when you were (N) years old. Can you tell me about your treatment at first – what was it like for you? (Prompt – what went well? What went badly? What would you have liked to change about the way your treatment went?

**Explain the concept of treat to target:**

- This study is part of a bigger project which we hope to do in the future. We plan to run a clinical study where we compare two different ways to treat **new** Lupus patients (in the first year). We know that different doctors have their favorite ways to treat Lupus. In this bigger project, patients would either be ‘treated to target’ by following a set plan, or treated in the usual way that their doctor usually treats Lupus.
- Treat to target involves:
- Setting a specific goal [doctors call this a target], that shows that a patient Lupus is feeling better
- Looking about once per month, to see how close or far away from the goal the patient is
- Changing the medication plan quickly if the goal is far away
- This diagram shows what a treat to target study would look like (show the plain diagram) – an annotated version of the diagram is shown below to help with explanation of treat to target study

- What do you think about the treat to target group?
- (Prompt - Do you think they are coming to hospital too often?)
- (Prompt - Do you think their treatment is building up too quickly?)
- (Prompt – Do you think it would be useful to have a treatment goal that your doctor is aiming for?)
- What do you think about the usual care group?
- (Prompt - Would you be happy to be in the routine care group?)
- (Prompt – do you think 3 monthly is frequent enough to come to hospital? Although the patient would come more frequently if the doctor was worried about them)
- How would you change this study if you were advising the doctors doing it?
- Would you have liked to take part in this study? (Prompt – if yes, why? If no, why not?)
