# Supplementary material for: ‘It is good to have a target in mind’: qualitative views of patients and parents informing a treat to target clinical trial in juvenile-onset systemic lupus erythematosus
Source: Rheumatology (Oxford). 2021 Feb 25;60(12):5630–41. doi: 10.1093/rheumatology/keab173 (PMC8645274; doi:10.1093/rheumatology/keab173)
Supplement: keab173_Supplementary_Data [file keab173_supplementary_data.zip › keab173-suppl_data/rhe-20-2289-File005.docx]

Topic guide for parent’s qualitative interviews

**‘Living with Lupus’ - developing treatment targets and outcome measures with children and young people to TARGET LUPUS**

NOTE: Qualitative interviews will be conversational and tailored to the individual participant. So, this is only an outline of the topics and questions that will be covered and it will be updated in the light of the ongoing interviews and analysis. Parental interviews should be carried out before the interview with the child / young person where possible.

***First section – background and establishing rapport***

*This first bit of the interview is all about your child’s initial diagnosis of Lupus.*

- What hospital is your [child name] treated at?
- When was [child’s name] diagnosed with Lupus?
- How did [child’s name] Lupus first start?
- How long did it take to find out what was wrong with [child’s name]?

***Second section – exploring what it means to patients with Lupus to ‘be well’ and comparing this to medical definitions of being in a ‘low disease activity state’***

*This part of the interview is to explore how [child’s name] feels when they are ‘well’ and ‘un-well’?*

- Since [child’s name] was diagnosed with Lupus, has there been a time when [child’s name] has felt well again well?
- How long after diagnosis did it take for [child’s name] to first feel well?
- Roughly, how long did [child’s name] feel well for?
- When [child’s name] is ‘well’ (or has a good day), does her/his Lupus go away? If no, what residual symptoms or signs of Lupus does [child’s name] have?
- Are there any features of Lupus that just won’t go away, no matter what medications [child’s name] tries?
- Since being diagnosed with Lupus, do you think [child’s name] has ever felt completely back to normal? (Here we are trying to get at whether they feel they have been in complete remission)
- Are there any symptoms of Lupus that you or [child’s name] don’t even bother mentioning to the doctor/anyone as you don’t think anything can be done about them?
- What is [child’s name] lupus like when it is at its worst? What is a bad day like?
- [This question relates to the existing definition of LDAS] - Would you class [child’s name] as ‘not perfect but fairly well’ if they had any of the following symptoms / signs of lupus?
  - Arthritis (PROMPT – 2 or more joints with pain or swelling)
  - Myositis (PROMPT - muscle aching or weakness)
  - A rash
  - Alopecia (PROMPT – hair loss)
  - Ulcers affecting you mouth or nose
  - Pain in their chest
  - A fever (PROMPT – temperature of more than 38^o^C)
- If a Lupus patient was described as being in a ‘low disease activity state’ where their lupus isn’t very active, but they are not completely back to what they were like before they had Lupus, what symptoms / signs of Lupus would you still expect them to have?
- When [child’s name] is ‘fairly well’ but not perfect, considering all the ways that Lupus affects her/him, how would you rate how [child’s name] is by placing a single mark on this line. [SHOW A PICTURE OF THE 0-10 SCALE BELOW].

Very well 0
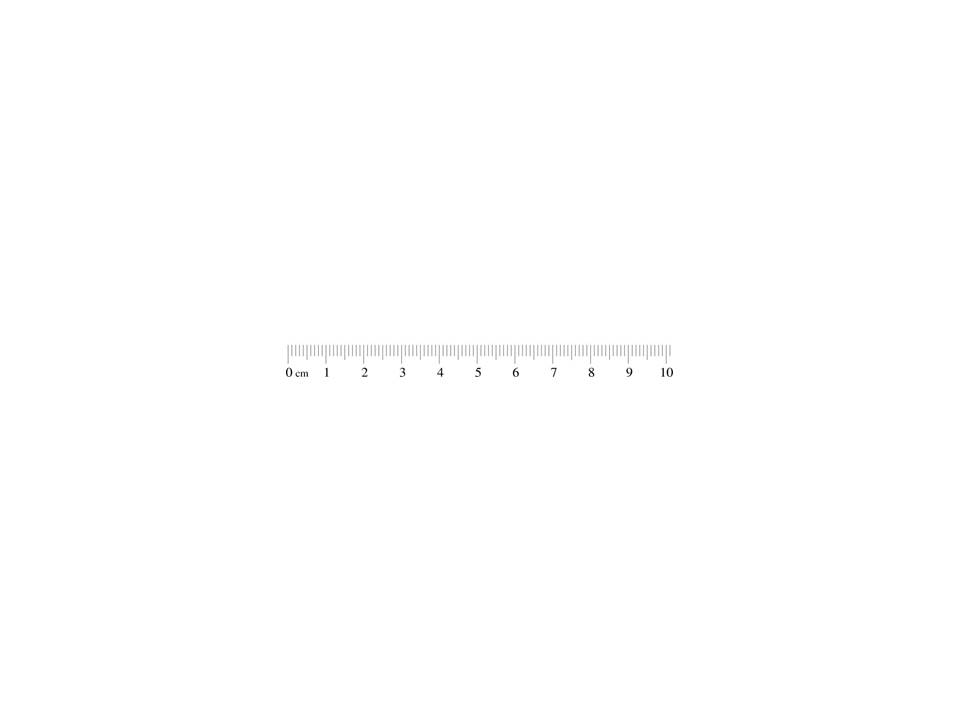
 10 Very poor

- At the times when [child’s name] has ‘felt well’, can you remember what treatments she/he was on?
  - Hydroxycholoquine – YES / NO
  - Mycophenolate mofetil – YES / NO
  - Azathioprine – YES / NO
  - Methotrexate – YES / NO
  - Rituximab – YES / NO
  - Cyclophosphamide – YES / NO
  - Any other treatments to dampen down the immune system?
- How much steroid medicine (prednisolone) has [child’s name] tended to be on when she/he has felt well? (At that time can you remember roughly how much she/he weighed so that we can work out the dose according to their weight, if not can you remember how old they were at the time, and we work out an average weight for their age?)

***Third section – patient reported outcome measures***

*In this section I would like to ask what you think of different questionnaires that can be used to monitor how [child’s name] is doing over time. When you are filling in the questionnaire it would be useful if you could say everything that you are thinking out loud, so that Sarah can get an idea of what you think and feel about the different questions. [NOTE – during periods of silence, remind the participant to keep thinking aloud].*

(Questionnaires to be confirmed following detailed literature review but anticipated to be at least two parent report questionnaires relating to HRQOL and two fatigue measures. For each tool we are interested in **a) respondent burden** (time to complete the questionnaire, the interviewer will record the time it takes to complete the questionnaires without making it too obvious, so as not to put the participant under pressure to complete the questionnaires quickly), **b) feasibility** (are they able to complete all questions, what proportion of the questions are left unanswered, why are they left unanswered e.g. can’t understand to question, not relevant to them, other), **c) expected answers when well** (when they are well do they think they would have a perfect score for all items or do they think there are some items where they still wouldn’t score perfectly).

For each questionnaire:

- Can you have a go at completing this questionnaire. Try to complete all of the questionnaire. If there are questions that you don’t understand / can’t complete, leave them blank and we will discuss them at the end. I will get on with some work in the meanwhile.
- [NOTE - At the end, after completing all questionnaires go through any questions left blank or that the participant found confusing].
- Thinking about a time when [child’s name] has been well, would you have given a perfect score for all items, or do you think there are some items where you wouldn’t have scored them perfectly? Even when [child’s name] is well, are there some activities mentioned in the questionnaire that she/he would still struggle with?
- Which HRQOL questionnaire do they prefer, and what makes you prefer that one?

Which fatigue measure do they prefer, and what makes you prefer that one?

***Fourth section – experiences of steroid treatment and reflecting on the adult glucocorticoid toxicity index***

*One of the types of medicine that [child’s name] will have been given is called a steroid (for example prednisolone or the intravenous version which is called methylprednisolone or methyl pred for short). This part of the interview is about those types of treatment.*

- Do you like or dislike steroid treatment? PROMPT – what makes you like or dislike them? Is there anything in particular that you like or dislike?
- What types of steroid has your child had in the past? (PROMPTS – oral, intravenous)
- Has [child’s name] doctors struggled to reduce the dose of her/his steroid treatment?
- Has [child’s name] had any side effects from steroids?
- What have been the worst side effects [child’s name] has had from steroids?
- Would you say that how [child’s name] feels depends on the dose of the steroid they are on? Is there a dose that makes them feel good? A dose that makes them feel bad?
- When [child’s name] has been on a lot of steroid in the past, have you wished that she/he could come off steroids (or reduce them) quicker than they were been able to?
- Would reducing the steroid dose you are on be a good treatment target?

**Fifth section – exploring parental experience of medical treatment and eliciting some preliminary opinions on a Lupus treat to target study**

In this final section, I would like to find out about the medical treatments [child’s name] has had. How you have felt about these treatments and the way [child’s name] care went in the first year after they were diagnosed with Lupus.

I would also like to explore what you think about the idea of a medical study that doctors would like to carry out, to find out how children and young people with Lupus should be treated. [Note - Explain/reassure before and afterwards, as necessary, that this is only an imaginary medical study at the moment. We are not actually requesting participation from [child’s name].

**Exploring the parents experience of medical treatment**

- Tell me about the treatment [child’s name] received in the first year after they were diagnosed with lupus? What was it like? (Prompts – what went well? What went badly? What would you have liked to change about the way [child’s name] treatment went?)
- Can you remember roughly how often [child’s name] was seen in clinic in the first year? Do you feel that they were seen frequently enough?
- Can you remember which treatments [child’s name] received in the first year after being diagnosed with lupus?
- How many drugs did you have to try until [child’s name] lupus got under control? Were you happy with how long each drug was tried before increasing the dose or stating another treatment?
- Did you or [child’s name] complete any questionnaires on health related quality of life, fatigue or steroid side effects? (prompt – child, parent or both)

**Explain the concept of treat to target:**

- This study is part of a bigger project which ultimately aims to develop a clinical study comparing two different ways to the treatment of **new** Lupus patients (in the first year). We know that different doctors have their favorite ways to treat Lupus. In this bigger project, patients would either be ‘treated to target’ by following a set plan, also known as a protocol, or treated in the usual way that their doctor usually treats Lupus.
- Treat to target is a treatment approach that involves:
  - Setting a specific goal [doctors call this a target], that shows that a patient Lupus is feeling better
  - Looking about once per month, to see how close or far away from the goal the patient is
  - Changing the medication plan quickly if the goal is far away
- This diagram shows what a treat to target study would look like (show the plain diagram) – an annotated version of the diagram is shown below to help with explanation of treat to target study

- Do you have any questions or comments about the proposed medical study?
- What do you think about the treat to target group?
- (Prompt - Do you think they are coming to hospital too often or that this would be ok?)
- (Prompt - Do you think their treatment is building up too quickly or that it would be ok?)
- (Prompt – Do you think it would be useful to have a treatment goal that your doctor is aiming for?)
- What do you think about the usual care group?
- (Prompt - Would you be happy to be in the routine care group?)
- (Prompt – do you think about 3 monthly is frequent enough to come to hospital? Although they would come more frequently if the doctor was worried about them)
- How would you change this study if you were advising the doctors doing it?
- Would you have liked [child’s name] to take part in this study? (Prompt – if yes, why? If no, why not?)
