# Supplementary material for: ‘It is good to have a target in mind’: qualitative views of patients and parents informing a treat to target clinical trial in juvenile-onset systemic lupus erythematosus
Source: Rheumatology (Oxford). 2021 Feb 25;60(12):5630–41. doi: 10.1093/rheumatology/keab173 (PMC8645274; doi:10.1093/rheumatology/keab173)
Supplement: keab173_Supplementary_Data [file keab173_supplementary_data.zip › keab173-suppl_data/rhe-20-2289-File006.docx]

**Supplementary data S4 – Standard explanation use during interviews of the concept of treat to target**

*In a treat to target study the doctor would assess disease activity in a more structured way (assessing for the target) looking at symptoms/signs/blood/urine results and compare what you are like today to what you were like at your last visit (with fixed time points between study visits). If there hasn’t been a sufficient improvement in disease activity target then we would either increase the dose of the treatment, add a new treatment or change the treatment.*

*This structured approach is already used in adults with rheumatoid using a target which involves counting the numbers of joints involved. It is also used in the treatment of high blood pressure where you aim for a certain blood pressure. In lupus it is slightly more complicated as it the disease can affect a number of different organs. In a Lupus treat to target study, there would therefore have to be an overall score which looks at all of the different organs that can be affected by lupus and also blood and urine results. The doctor would measure this target every time they see the patient.*
