# Supplementary material for: ‘It is good to have a target in mind’: qualitative views of patients and parents informing a treat to target clinical trial in juvenile-onset systemic lupus erythematosus
Source: Rheumatology (Oxford). 2021 Feb 25;60(12):5630–41. doi: 10.1093/rheumatology/keab173 (PMC8645274; doi:10.1093/rheumatology/keab173)
Supplement: keab173_Supplementary_Data [file keab173_supplementary_data.zip › keab173-suppl_data/rhe-20-2289-File007.docx]

**Supplementary Figure 5. Diagram shown to families to illustrate what a hypothetical T2T study could involve.**
